# Effect of Manufacturing on the Transverse Response of Polymer Matrix Composites

**DOI:** 10.3390/polym13152491

**Published:** 2021-07-28

**Authors:** Sagar P. Shah, Marianna Maiarù

**Affiliations:** Department of Mechanical Engineering, University of Massachusetts Lowell, Lowell, MA 01854, USA; sagar_shah@student.uml.edu

**Keywords:** thermoset composites, process modeling, curing, damage mechanics, finite elements

## Abstract

The effect of residual stress build-up on the transverse properties of thermoset composites is studied through direct and inverse process modeling approaches. Progressive damage analysis is implemented to characterize composite stiffness and strength of cured composites microstructures. A size effect study is proposed to define the appropriate dimensions of Representative Volume Elements (RVEs). A comparison between periodic (PBCs) and flat (FBCs) boundary conditions during curing is performed on converged RVEs to establish computationally efficient methodologies. Transverse properties are analyzed as a function of the fiber packing through the nearest fiber distance statistical descriptor. A reasonable mechanical equivalence is achieved for RVEs consisting of 40 fibers. It has been found that process-induced residual stresses and fiber packing significantly contribute to the scatter in composites transverse strength. Variation of ±5% in average strength and 18% in standard deviation are observed with respect to ideally cured RVEs that neglect residual stresses. It is established that process modeling is needed to optimize the residual stress state and improve composite performance.

## 1. Introduction

Fiber-reinforced polymer matrix composites (PMCs) are widely used in structural applications due to their high strength and lightweight attributes and their superior fatigue and corrosion resistance [1,2,3,4]. Such composites are manufactured by curing the matrix, that surrounds the interspersed fibers, under high temperature and pressure conditions. During this process, cure-induced volumetric shrinkage of the matrix, mismatch in the thermo-mechanical properties between the fibers and the matrix, and thermal gradients due to exothermic reactions lead to significant residual stress generation and, at times, damage accumulation [5]. Random and off-axial fiber architecture, introduced during manufacturing, alters the composite stress state which may further contribute to the onset and evolution of damage [6,7,8,9,10]. Internal defects such as voids and microcracks may also occur [11,12,13,14,15]. Such defects can degrade the in situ matrix properties significantly and therefore, affect the composite mechanical response during subsequent load applications [16,17,18,19,20,21]. Despite these significant research contributions, a knowledge gap exists on the evolution of these process-induced uncertainties and their influence on the composite mechanical response such as transverse tensile [17,18,19,20,22,23] and compressive response [17,24]. It is paramount to address these knowledge gaps with novel and reliable process modeling approaches.

Computational micromechanics is an emerging field that leverages the advances in finite element methods (FEM) and the ever-increasing computing capabilities to accurately predict the microscale response of composite representative volume elements (RVEs) when subjected to various thermo-mechanical boundary conditions [16,17,18,19,20,21,23,24,25,26,27,28,29]. Within this framework, process-induced residual stress generation and damage accumulation can be obtained, and their influence on the bulk composite properties can be investigated by means of virtual curing and mechanical loading simulations of RVEs. Computational micromechanics offers a sophisticated, high-fidelity solution for evaluating process-induced residual stresses. The numerical framework presents several key advantages over the classic homogenization techniques and experiments [28]. The influence of the RVE geometry and the spatial distribution of fibers, which affect the stress state of the microstructure, can be taken into account. Additionally, the prediction of the stress and strain microfields throughout the microstructure allow precise estimations of stress concentration, damage initiation, and propagation during curing and subsequent mechanical loading. Several numerical studies have been performed to characterize the generation of process-induced residual stresses and evaluate their effect on the processed composite performance [16,17,18,19,20,21,23,24,25,26,27,29]. These studies most commonly utilize the phenomenological cure kinetic model, introduced by Kamal [30], along with the Fourier’s heat transfer law to define the progression of cure, heat generation, and heat conduction in the PMCs during cure. Coupled thermo-mechanical analysis with appropriate constitutive models have been used to predict residual stresses in composite microstructures including incremental elasticity model [31,32], linear mixing rule based on composite laminate plate theory [33], instantaneous linear elastic models [16,17,18], CHILE model [24], linear and nonlinear visco-elastic models [34,35,36,37,38], elasto-plastic model [23,26], network-based model [19,20,22,39,40], columnar model [29], and analytical models [41]. Some studies have investigated the effect of process-induced residual stresses on the bulk composite response under different loading scenarios. For instance, D’Mello et al. [19,20,22] quantified this effect on the transverse tensile response of carbon fiber-reinforced PMCs using RVEs with regular and irregular packing. Maiarù et al. [17] compared the effects of residual stresses on IM7/Epon862 RVEs under different loading conditions based on multi-fiber irregular arrangement. Maiarù [18] also investigated the influence of matrix fracture properties on the transverse response of virtually cured IM7/Epon862 RVEs based on random fiber distribution. These studies demonstrated that process-induced residual stresses affected the in situ matrix properties and the effective stiffness and strength of the composite. More recently, Danzi et al. [23] integrated the network model proposed by Heinrich et al. [40] to estimate matrix shrinkage and elastic property evolution during cure with damageable elasto-plastic constitutive relations, to emphasize on the importance of the curing process in damage analysis of composites. On similar lines, Hui et al. [24] developed an analytical numerical framework and investigated the effect of residual stresses on the transverse compressive response of IM7/8552 RVEs under quasi-static and dynamic test conditions. Alternatively, inverse process modeling analyses can be carried out to obtain the in-situ matrix response with relative ease and at low cost. Such strategies employ experimental procedures, including uniaxial tension [42] and compression tests [43], torsion tests [44], to measure the composite response. The in situ matrix properties are then extracted from the composite response using analytical and constitutive relationships as reported in [42,43,44]. These strategies are easy to implement, cost-effective, and provide relatively accurate results.

**Figure 1:**
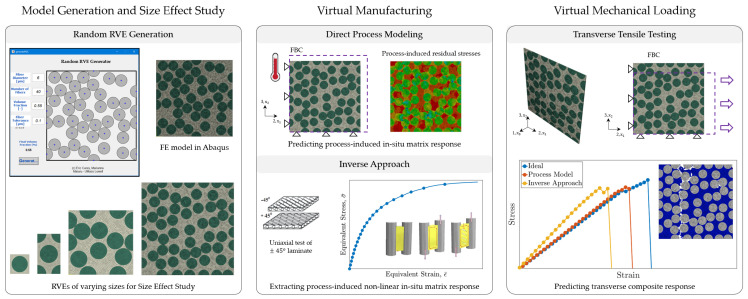
Schematic of the virtual analysis procedure [42].

The objective of this study is to implement the aforementioned direct and inverse process modeling approaches to quantify the effect of the manufacturing process on the transverse response of multi-fiber, randomly distributed composite RVEs. Transverse property predictions from the two approaches are compared and their effectiveness is evaluated. For this purpose, a virtual analysis procedure is established, as detailed in Section 2 and schematically presented in Figure 1. Composite microstructures of varying sizes, comprising of a random dispersion of IM7 carbon fibers embedded in an EPIKOTE^TM^ Resin MGS^®^ RIMR 135/ EPIKURE^TM^ Curing Agent MGS^®^ RIMH 1366 (here onwards referred to as RIM R135/H1366 for brevity) epoxy matrix, are modeled in commercial finite element (FE) software Abaqus, The modeling details are discussed in Section 2.1. The generated RVEs are first, virtually manufactured to determine the evolution of process-induced in situ matrix response. The following two approaches are considered in this study:

(a) Direct process modeling: FE-based approach that predicts the instantaneous in situ matrix property evolution and residual stress generation as a function of the processing conditions and the degree of cure ϕ. Its implementation is detailed in Section 2.2.1.
(b) Inverse approach: experiment-based approach that extracts the process-induced, nonlinear in situ matrix properties from the uniaxial tensile response of a ±45${}^{{}^{\circ}}$ laminate. The detailed procedure is reported by [42], its summary is presented in Section 2.2.2.

Subsequently, the virtually manufactured RVEs are subjected to transverse mechanical loading to predict the transverse composite stiffness $E_{22}^{+}$ and strength $\sigma_{22}^{+}$, the details of which are reported in Section 2.3. In order to quantify the effect of manufacturing on the transverse composite response, the predictions from the two approaches are compared to a third case which assumes an initial stress-free state and neglects the effect of the processing conditions on the in situ matrix properties. In doing so, a size effect study is carried out to establish mechanical equivalence based on the transverse property predictions and define appropriate RVE size. Furthermore, statistical analyses are carried out to provide direct correlation between the mechanical properties and random fiber packing in the RVE. The analysis results are discussed in Section 3, and the main conclusions are drawn in Section 4.

## 2. Virtual Analysis Procedure

The virtual analysis procedure presented in Figure 1 is discussed in further detail in the present section.

### 2.1. Microscale Computational Model

Computational micromechanical analyses of composite microstructures are carried out on multi-fiber, randomly packed RVEs. In order to accurately predict the bulk mechanical properties, these RVEs must be sufficiently large, such that they can capture the characteristic features that influence the stress state and failure within a ply including random spatial distribution of fibers, local variation in the fiber volume fraction, relative fiber proximity, and stress concentration within the RVEs [10,24,45,46,47]. At the same time, the RVEs must be computationally efficient. To this end, a size effect study is proposed to determine the appropriate RVE size for accurate property prediction at a reasonable computational cost. Several different realizations of composite microstructures of varying sizes are virtually analyzed following the procedure described below. Results are analyzed in terms of the mean and standard deviation of the transverse properties against the computational cost of the analysis to determine the representative size of the microstructure. A statistical analysis is carried out on the RVEs by utilizing statistical descriptors based on the spatial arrangement of fiber to justify the choice of the RVE size and provide direct correlation between the mechanical properties and the RVE fiber packing.

Several strategies to generate random RVEs can be found in the literature [9,10,48,49,50,51,52,53,54,55,56,57,58,59]. For the present study, a random RVE generator is developed in-house with MATLAB and used to generate FE models of various sizes and random fiber distribution in Abaqus [60]. For a given fiber diameter $d_{f}$, fiber volume fraction $v_{f}$, and number of fibers $n_{f}$, the algorithm generates randomly distributed fiber centers. The fiber coordinates are then imported into Abaqus where the RVE is modeled, discretized and appropriate boundary conditions are applied [61]. This process is automated through Python scripting.

**Figure 2:**
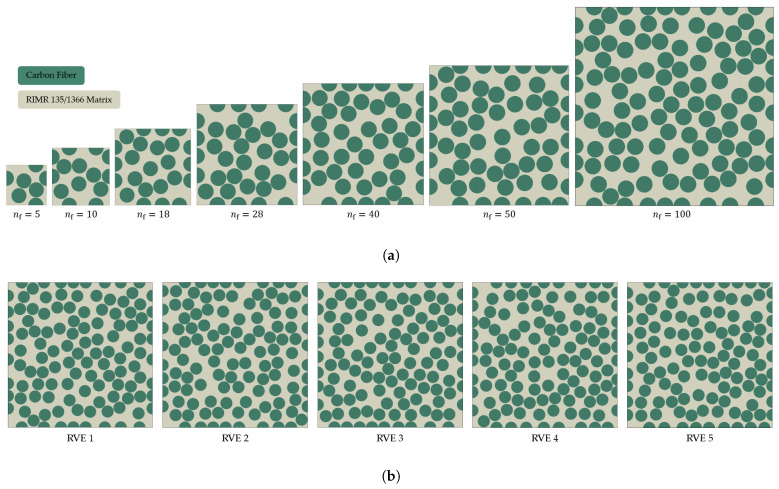
Various realizations of the composite microstructures generated by the random RVE generator: (**a**) shows the several sizes of RVE considered in this study and (**b**) show five distinct realizations of a 100-fiber RVE.

The composite RVEs, comprising of interspersed carbon fibers embedded ($d_{f}=6\;\mu m$) in a RIM R135/H1366 epoxy matrix with an average fiber volume fraction $v_{f}=0.55$, are generated by utilizing the random RVE generator. The bonding between the fiber and the matrix is considered perfect. Several sizes of the RVEs ($1\leq n_{f}\leq 100$), illustrated in Figure 2a, are considered for the size effect study. Five distinct realizations with random fiber distribution are analyzed for each value of $n_{f}$, with the exceptions of $n_{f}=1,2$ which represent a square and hexagonal packing, respectively. Five realizations of a 100 fiber RVE with random fiber distribution are presented in Figure 2b. The fibers are modeled as transversely-isotropic linear-elastic solids, the thermo-mechanical properties for which are summarized in Table 1. The matrix is modeled as isotropic material. The thermo-mechanical properties for a fully cured epoxy matrix (ϕ=1) are listed in Table 2. The matrix material behavior is defined with user-written subroutines: UMATHT and UMAT for direct process modeling (Abaqus/STANDARD) and VUMAT for virtual mechanical loading analysis (Abaqus/EXPLICIT), the implementations of which are detailed in the following sections [61]. Each FE model is meshed with C3D8RT elements (eight node brick elements with reduced integration and temperature degrees of freedom) [61].

### 2.2. Virtual Manufacturing

This section details the virtual manufacturing analysis of composite microstructures to determine the evolution of the process-induced in situ matrix properties using direct and inverse process modeling approaches.

#### 2.2.1. Direct Process Modeling

The present approach directly predicts the process-induced in situ matrix response and residual stresses as a function of the processing conditions and the degree of cure ϕ. This method accounts for two key aspects associated with the manufacturing process: (a) kinetic progression of the cure which is defined by ϕ and (b) the evolution of the in situ thermo-mechanical matrix properties which leads to residual stress generation.

In this study, an autocatalytic phenomenological semi-empirical kinetic model (Equation (1)), developed by Kamal [30], is used to define the progression of the cure for a given cure cycle.

(1) $$\frac{d\phi }{dt}=\left[A_{1}\;\exp\left(-\frac{\Delta E_{1}}{RT}\right)+A_{2}\;\exp\left(-\frac{\Delta E_{2}}{RT}\right)\phi^{m}\right]\left(1-\phi^{n}\right)$$

where *T* is the temperature, *R* is the gas constant, $\Delta E_{1}$ and $\Delta E_{2}$ are activation energies, $A_{1}$ and $A_{2}$ are frequency-like constants, and *m* and *n* are exponents. These kinetic constants for the RIM R135/H1366 epoxy are determined through experiments and summarized in Table 3.

**Figure 3:**
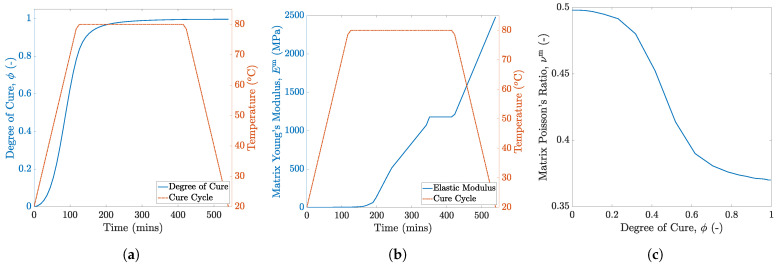
Thermo-mechanical property inputs for RIMR R135/H1366: (**a**) evolution of the degree of cure with temperature and time, (**b**) matrix Young’s modulus $E^{m}$, and (**c**) matrix Poisson’s ratio $\nu^{m}$.

**Figure 4:**
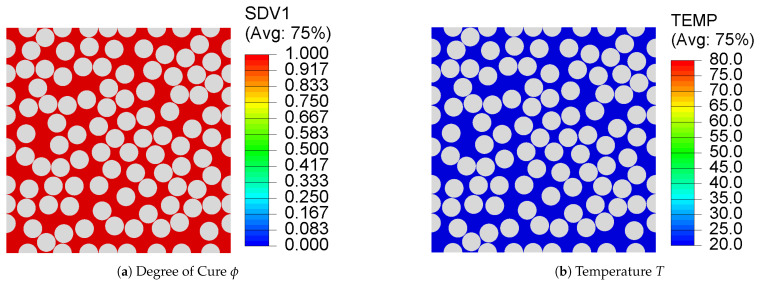
Contour plots of (**a**) degree of cure ϕ and (**b**) temperature in a 100-fiber RVE at the end of the prescribed cure cycle indicating a uniform cure evolution and temperature distribution.

A coupled temperature-displacement analysis is carried out in Abaqus/STANDARD with user-subroutine UMATHT, which simultaneously solves the autocatalytic kinetic model (Equation (1)) and the Fourier heat transfer model (Equation (2)) in 3-dimension to predict the progression of cure and the temperature distribution, as a result of the exothermic heat of reaction.

(2) $$\rho c_{p}\frac{dT}{dt}=k_{i}\frac{d^{2}T}{dt^{2}}+\frac{dq}{dt}\;\;\;\;\mathrm{where},\;\;\;\;\frac{dq}{dt}=\rho H_{T}\frac{d\phi }{dt}$$

where ρ is the density, $c_{p}$ is the specific heat capacity, $k_{i}$ is the thermal conductivity, *q* is the instantaneous exothermic heat released during curing, and $H_{T}$ is the total heat of reaction. The temperature profile used for virtual manufacturing of composite microstructures and the computed degree of cure ϕ are presented in Figure 3a. Given the length scale, no variations are observed in degree of cure for the 100 fiber RVE as shown in Figure 4a, that is, the temperature distribution is uniform and no internal thermal gradients are observed at the microscale. This is evident from Figure 4b.

During curing, the chemo-rheological and thermo-mechanical properties of the in situ matrix evolve with the progression of cure. These properties for the RIM R135/H1366 epoxy system are characterized in-house and presented in Figure 3. The post-gelation coefficient of thermal expansion $\alpha^{m}$ and chemical shrinkage coefficient $\beta^{m}$ are constants and listed in Table 2. Once the degree of cure is determined for a given time increment, the instantaneous material properties associated with that cure state are computed by user-subroutine UMAT. An instantaneous linear-elastic constitutive model (Equation (3)), introduced by Maiarù [17], is used to predict the residual stress generation due to the development of the matrix elastic modulus and the chemical and thermal strains experienced by the matrix.

(3) $$\sigma_{i}\left(t\right)=\left[C_{\mathrm{ij}}\left(t\right)\right]\left[\epsilon_{j}^{t}\left(t\right)-\left(\epsilon_{j}^{\mathrm{th}}\left(t\right)+\epsilon_{j}^{\mathrm{sh}}\left(t\right)\right)\delta_{j}\right]\;\;\;\;\mathrm{where},\;\;\left\{\begin{array}{cc} \delta_{j}=1 & \mathrm{if}\;j\;=\;1,2,3\\ \delta_{j}=0 & \mathrm{if}\;j\;>\;3 \end{array}\right.$$

where i and j are Voigt notation indices, $\epsilon_{j}^{t}\left(t\right)$, $\epsilon_{j}^{\mathrm{th}}\left(t\right)$, and $\epsilon_{j}^{\mathrm{sh}}\left(t\right)$ are the total, thermal, and chemical shrinkage strains, respectively, $C_{\mathrm{ij}}\left(t\right)$ is the stiffness matrix as a function of the time of cure and $\sigma_{i}\left(t\right)$ is the accumulated residual stress. In this study, the curing matrix is prescribed a constant strength and fracture toughness as listed in Table 2.

**Figure 5:**
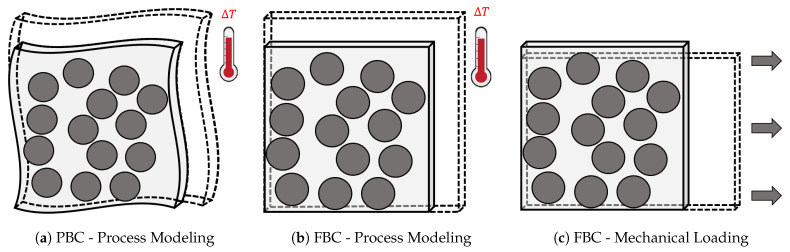
Illustrations of various boundary conditions applied to the RVEs during the analyses: (**a**) Periodic Boundary Conditions (PBC) and (**b**) Flat Boundary Conditions (FBC) for direct process modeling analysis, and (**c**) Flat Boundary Conditions for mechanical loading analysis.

The fiber and the matrix in the composite microstructure tend to expand and contract due to temperature change in different phases of the cure cycle during manufacturing. Furthermore, the matrix contracts due to chemical shrinkage as cure progresses. In order to account for these volumetric changes in the FE model, two sets of boundary conditions are considered in this study: (a) Periodic Boundary Conditions (PBCs) and (b) Flat Boundary Conditions (FBCs). PBCs are often applied to composite microstructures in concert with the assumption that an RVE is a small volume within an infinite medium. Consequently, PBCs ensure that the microstructures deform in a periodic manner as illustrated in Figure 5a. However, the implementation of PBCs in Abaqus requires defining a large number of *EQUATION cards which is computationally expensive [61]. FBCs are a special case of PBCs where the microstructure is allowed contract and expand during cure with one constrain—the faces of the RVE must remain flat at all times. FBCs deformations are illustrated in Figure 5b. The number of *EQUATION cards required to define FBCs are significantly less compared to PBCs. Consequently, the computational cost associated with FBC is also considerably low.

**Figure 6:**
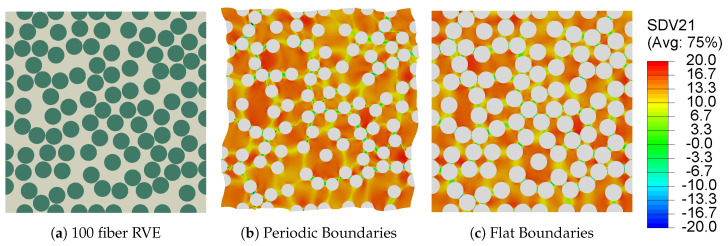
Contour plots illustrating the process-induced residual stresses (maximum principal) and the final deformed shape (deformation magnified 7× for visualization) of (**a**) 100 fiber RVE at the end of the cure when subjected to (**b**) Periodic Boundary Conditions and (**c**) Flat Boundary Conditions.

In order to illustrate the effect of boundary conditions on the numerical predictions during curing, a 100-fiber RVE (Figure 6a) is virtually manufactured with the direct process modeling approach. The temperature profile presented in Figure 3a is used to cure the RVE, first with PBC and then with FBC. The residual stresses generated in each case are then compared in Figure 6b,c. Both set of boundary conditions yield the same residual stress build-up at the end of the cure cycle. Furthermore, it is observed that the periodic deformations in the RVE with PBCs are negligible (magnified 7× for visualization). Therefore, for the sake of computational efficiency, FBCs are used for all subsequent analyses [17].

#### 2.2.2. Inverse Approach

The following approach is based on the assumption that the effect of manufacturing on the in situ matrix can be accounted for indirectly by extracting the in situ matrix behavior from experiments. The nonlinear in situ matrix behavior is determined from uniaxial tension test of composite laminates as detailed in [42].

**Figure 7:**
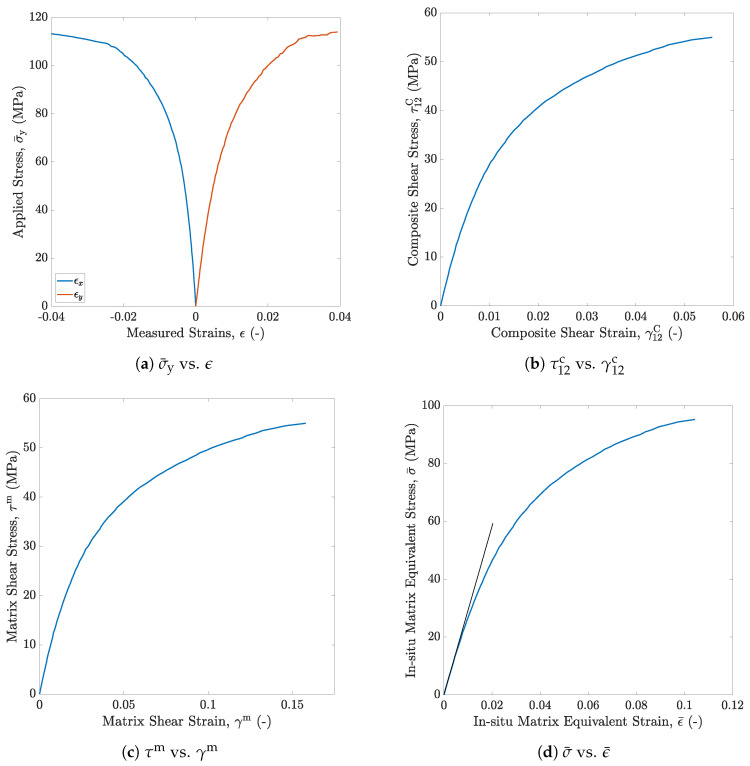
Plot of (**a**) applied stress versus measured strain from the uniaxial test, (**b**) shear response of the composite laminate, (**c**) shear response of the in situ matrix, and (**d**) equivalent stress versus strain response of the in situ matrix.

In this study, specimens conforming to ISO 527-4 Type 2 geometry are cut out from a ±45${}^{{}^{\circ}}$ laminate and tested in tension under quasi-static conditions at TPI Composites. The applied stress ${\bar{\sigma }}_{y}$ is monitored throughout the test, the axial and transverse strains ($\epsilon_{y}$ and $\epsilon_{x}$) are recorded with back to back strain gages attached at the center of the specimen. The applied stress versus measured strain plot is presented in Figure 7a. For a symmetric laminate subjected to axial stress ${\bar{\sigma }}_{y}$, the composite shear stress $\tau_{12}^{c}$ and shear strain $\gamma_{12}^{c}$ are then computed using,

(4) $$\tau_{12}^{c}=\pm \frac{{\bar{\sigma }}_{y}}{2}\;\;\;\;\;\;\mathrm{and}\;\;\;\;\;\;\gamma_{12}^{c}=-\left(\epsilon_{x}-\epsilon_{y}\right)$$

The $\tau_{12}^{c}$ versus $\gamma_{12}^{c}$ plot obtained using Equation (4) is shown in Figure 7b. The highly nonlinear nature of the shear response is attributed to micro-cracking observed in the resin in between the fibers, which adds to the global compliance [42].

Once the composite shear stress and shear strain are obtained from the tensile test, the secant shear modulus of the composite laminate $G_{12}^{c}$ is calculated as

(5) $$G_{12}^{c}=\frac{\tau_{12}^{c}}{\gamma_{12}^{c}}=\frac{{\bar{\sigma }}_{y}}{2(\epsilon_{x}-\epsilon_{y})}$$

Here, it is assumed that Equation (5) is valid through the nonlinear regime of the shear stress-shear strain response as well [42]. The secant shear modulus $G_{12}^{c}$ is computed as a function of the shear stress $\tau_{12}^{c}$. Thus, knowing the fiber volume fraction ($v_{f}=0.49$) and the fiber shear modulus ($G_{12}^{f}=30\;\mathrm{GPa}$), the in situ matrix shear modulus $G^{m}$ is computed using Concentric Cylinder Assemblage (CCA) model:

(6) $$G_{12}^{c}=\frac{G_{12}^{f}\left(1+v_{f}\right)+G^{m}\left(1-v_{f}\right)}{G_{12}^{f}\left(1-v_{f}\right)+G^{m}\left(1+v_{f}\right)}G^{m}$$

Assuming that the shear stress sustained by the fiber ($\tau_{12}^{f}$), the matrix ($\tau^{m}$), and the composite ($\tau_{12}^{c}$) are same [42], the in situ matrix shear strain $\gamma^{m}$ is computed as

(7) $$G^{m}=\frac{\tau^{m}}{\gamma^{m}}$$

The plot for the in situ matrix shear stress versus shear strain is presented in Figure 7c.

Neglecting the effect of normal stresses, the J_2_ deformation theory of plasticity,

(8) $$\bar{\sigma }=\sqrt{3J_{2}}=\sqrt{3S_{\mathrm{ij}}S_{\mathrm{ij}}}=\sqrt{3}\tau^{m}\;\;\;\;\;\;\mathrm{and}\;\;\;\;\;\;{\bar{\epsilon }}_{p}=\sqrt{\frac{2}{3}\epsilon_{\mathrm{ij}}^{p}\epsilon_{\mathrm{ij}}^{p}}=\frac{\gamma_{p}^{m}}{\sqrt{3}}$$

is used to determine the in situ matrix equivalent stress $\bar{\sigma }$ versus equivalent plastic strain ${\bar{\epsilon }}_{p}$ relation. Here, the total strains are decomposed into elastic and plastic components, $\epsilon_{\mathrm{ij}}=\epsilon_{\mathrm{ij}}^{e}+\epsilon_{\mathrm{ij}}^{p}$. The in-situ matrix Young’s modulus required to compute the elastic strains is obtained from the in situ matrix shear modulus and Poisson’s ratio (ν=0.37). The in situ matrix equivalent stress versus equivalent strain curve is shown in Figure 7d.

A built-in elasto-plastic material definition is implemented in Abaqus/EXPLICIT to test RVEs modeled using the inverse approach [61]. The in situ matrix Young’s modulus and Poisson’s ratio define the linear elastic response (see Table 2), and the nonlinear inelastic behavior is defined by the in situ matrix equivalent stress $\bar{\sigma }$ versus equivalent strain $\bar{\epsilon }$ plot shown in Figure 7d.

### 2.3. Virtual Mechanical Loading

At the end of the virtual manufacturing step, the in situ matrix material is fully cured (ϕ=1) and its thermo-mechanical properties are established. Subsequently, the RVE is subjected to mechanical loading in transverse tension by prescribing a velocity boundary condition. FBCs, as illustrated in Figure 5c, are enforced to maintain periodicity during this step. The objective here is to compute the transverse composite stiffness $E_{22}^{+}$ and strength $\sigma_{22}^{+}$ of the virtually manufactured RVEs and quantify the effect of manufacturing on the transverse composite response.

**Figure 8:**
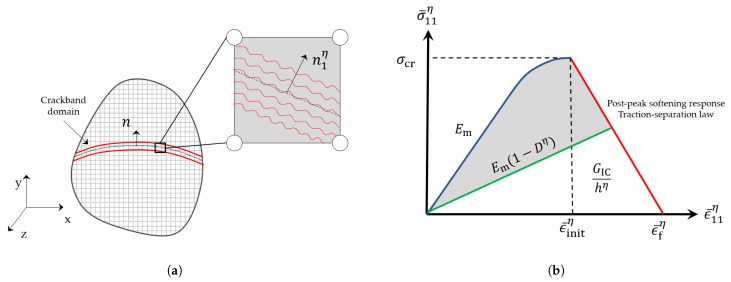
(**a**) Illustration of a crackband embedded in a discretized continuum; magnified inset displays the crackband orientation and smeared cracks within an element η, and (**b**) the progressive damage formulation in the principal frame based on the theory of Crackband.

For RVEs analyzed with direct process modeling approach, a progressive damage model based on the theory of Crackband [62] is used to model failure in the matrix material as illustrated in Figure 8. The maximum principal stress criterion is utilized to determine the failure initiation in the matrix. The traction-separation law, presented in Figure 8b and governed by the fracture energy, is employed to define the post-peak softening behavior of the damaging material once the critical fracture stress $\sigma_{\mathrm{cr}}$ is reached. The fracture energy is the strain energy released during the formation of new surfaces during crack growth and is assumed to be dissipated over the entire element. Monolithic materials, such as the matrix material modeled in FE, are locally subjected to pure mode I failure [63] and therefore, the critical mode I energy release rate $G_{\mathrm{IC}}$ is given by

(9) $$G_{\mathrm{IC}}=h^{\eta }\int_{0}^{{\bar{\epsilon }}_{f}^{\eta }}{\bar{\sigma }}_{11}^{\eta }\left({\bar{\epsilon }}_{11}^{\eta }\right)\;d\bar{\epsilon }$$

where ${\bar{\sigma }}_{11}^{\eta }$ and ${\bar{\epsilon }}_{11}^{\eta }$ are the maximum principal stress and strain values in element η, respectively; ${\bar{\epsilon }}_{f}^{\eta }$ is the value of ${\bar{\epsilon }}_{11}^{\eta }$, which corresponds to a zero stress state on the post-peak stress versus strain plot (see Figure 8b); and $h^{\eta }$ is the characteristic length of the element η that preserves mesh objectivity by prescribing a normalized value of $G_{\mathrm{IC}}$ for each element, such that $g_{\mathrm{IC}}^{\eta }=G_{\mathrm{IC}}/h^{\eta }$.

Once failure has initiated in element η (Figure 8a), the crackband orientation is fixed as the analysis progresses. Its orientation is given by a vector $n_{1}^{\eta }$ which is determined from the local principal stress state $\left\{{\bar{\sigma }}_{11}^{\eta },\;{\bar{\sigma }}_{22}^{\eta },\;{\bar{\sigma }}_{33}^{\eta }\right\}$. Subsequently, the element compliance $S^{\eta }$ is rotated to the fixed principal frame using the transformation matrix $Q^{\eta }$,

(10) $$Q^{\eta }=\left[n_{1}^{\eta }\;n_{2}^{\eta }\;n_{3}^{\eta }\right]\left[e_{1}\;e_{2}\;e_{3}\right]$$

where $n_{i}^{\eta }$ are the principal stress directions and $e_{i}$ are the unit base vectors (i=1,2,3). All the material degradation associated with the crackband evolution is enforced on the rotated compliance ${\bar{S}}^{\eta }$, the components of which are computed as

(11) $${\bar{S}}_{\mathrm{ijkl}}^{\eta }=Q_{\mathrm{wi}}^{\eta }\;Q_{\mathrm{xj}}^{\eta }\;S_{\mathrm{wxyz}}^{\eta }\;Q_{\mathrm{ky}}^{\eta }\;Q_{\mathrm{lz}}^{\eta }$$

The local, rotated strain state in the element $\left\{{\bar{\epsilon }}_{11}^{\eta },\;{\bar{\epsilon }}_{22}^{\eta },\;{\bar{\epsilon }}_{33}^{\eta }\right\}$ is used to compute the scalar damage factor in order to degrade the rotated compliance components.

(12) $${\bar{\epsilon }}_{\mathrm{ij}}^{\eta }=Q_{\mathrm{wi}}^{\eta }\;\epsilon_{\mathrm{wx}}^{\eta }\;Q_{\mathrm{xj}}^{\eta }$$

The scalar, damage factor $D^{\eta }$ is calculated using the rotated strains.

(13) $$D^{\eta }=1-\frac{\sigma_{\mathrm{cr}}}{E_{m}\left({\bar{\epsilon }}_{f}^{\eta }-{\bar{\epsilon }}_{\mathrm{init}}^{\eta }\right)}\left(\frac{{\bar{\epsilon }}_{f}^{\eta }}{{\bar{\epsilon }}_{11}^{\eta }}-1\right)$$

where ${\bar{\epsilon }}_{\mathrm{init}}^{\eta }$ is the value of ${\bar{\epsilon }}_{11}^{\eta }$ when the initiation criterion (${\bar{\sigma }}_{11}^{\eta }\geq \sigma_{\mathrm{cr}}$) is satisfied (Figure 8b), $E_{m}$ is the undamaged Young’s modulus of the matrix. The damage parameter can take values between zero and one, where $D^{\eta }=0$ means no damage has occurred. By contrast, a maximum damage level of one corresponds to a zero stress state on the post-peak stress versus strain plot (Figure 8b). Furthermore, healing is inadmissible. Once the damage factor is computed, the components of the rotated compliance matrix are degraded as

(14) $${\bar{S}}^{\eta }=\left[\begin{array}{cccccc} \frac{{\bar{S}}_{1111}^{\eta }}{1-D^{\eta }} & {\bar{S}}_{1122}^{\eta } & {\bar{S}}_{1133}^{\eta } & 0 & 0 & 0\\ {\bar{S}}_{1122}^{\eta } & {\bar{S}}_{2222}^{\eta } & {\bar{S}}_{2233}^{\eta } & 0 & 0 & 0\\ {\bar{S}}_{1133}^{\eta } & {\bar{S}}_{2233}^{\eta } & {\bar{S}}_{3333}^{\eta } & 0 & 0 & 0\\ 0 & 0 & 0 & \frac{{\bar{S}}_{1212}^{\eta }}{1-D^{\eta }} & 0 & 0\\ 0 & 0 & 0 & 0 & \frac{{\bar{S}}_{1313}^{\eta }}{1-D^{\eta }} & 0\\ 0 & 0 & 0 & 0 & 0 & {\bar{S}}_{2323}^{\eta } \end{array}\right]$$

The shear compliance ${\bar{S}}_{1212}^{\eta }$ and ${\bar{S}}_{1313}^{\eta }$ are degraded in addition to the normal compliance ${\bar{S}}_{1111}^{\eta }$ in the rotated frame to ensure that the faces of crack within the band normal to $n_{1}^{\eta }$ are free of all normal and shear traction when all of the crackband energy has been dissipated. After degrading the rotated compliance matrix, it is transformed back to the global frame to yield the new element compliance using

(15) $$S_{\mathrm{ijkl}}^{\eta }=Q_{\mathrm{wi}}^{-1^{\eta }}\;Q_{\mathrm{xj}}^{-1^{\eta }}\;{\bar{S}}_{\mathrm{wxyz}}^{\eta }\;Q_{\mathrm{ky}}^{-1^{\eta }}\;Q_{\mathrm{lz}}^{-1^{\eta }}$$

The progressive damage formulation is modeled in Abaqus/EXPLICIT solver with user-subroutine VUMAT [61]. The matrix strength $\sigma_{\mathrm{cr}}$ and a scaled-down fracture toughness $G_{\mathrm{IC}}$ corresponding to sub-micron length scale are prescribed to the material as listed in Table 2.

For the inverse approach, failure in the in situ matrix material is defined by the *TENSILE failure card available in Abaqus/EXPLICIT [61]. This criterion uses a pressure based failure criteria which requires the hydrostatic cut-off stress to determine failure initiation. For this study, the maximum hydrostatic stress value of $p_{\max}=43\;\mathrm{MPa}$ is used to define failure initiation. This value corresponds to the critical stress value of $\sigma_{\mathrm{cr}}=64.1\;\mathrm{MPa}$ in the matrix material and is determined from the material stress state {σ} when ${\bar{\sigma }}_{11}=\sigma_{\mathrm{cr}}$.

Following the procedure discussed in this section, the size effect study is performed for all three material definitions. All realizations of the FE model are virtually tested in transverse tension assuming appropriate initial stress state and material definition. The results from the virtual analysis and size effect study are discussed in the following section.

## 3. Results and Discussion

**Figure 9:**
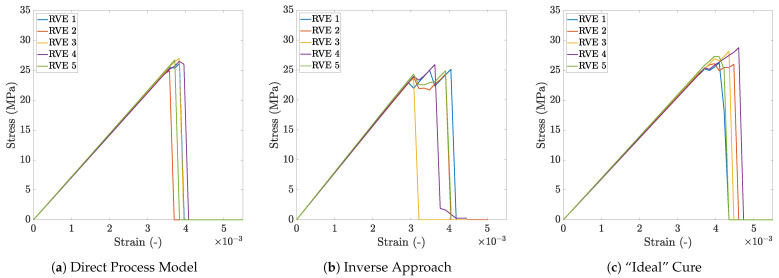
Plots of transverse tensile stress versus strain for 100 fiber RVEs obtained from (**a**) direct process modeling, (**b**) inverse approach, and (**c**) “ideal” cure analysis without process-induced residual stresses.

An investigation into the process-induced in situ matrix property evolution and residual stress generation, and their effect on the matrix-dominated transverse composite response, is carried out on randomly packed RVEs of varying sizes. Virtually manufactured RVEs, with direct and inverse process modeling approaches, are tested in transverse tension. The transverse property predictions from these two approaches are compared to an “ideal” cure case in order to quantify the influence of manufacturing on the composite response. For comparison, the stress versus strain plots of the 100-fiber RVEs, that are analyzed assuming appropriate material definitions, are presented in Figure 9. The comparison manifests up to 5% reduction in the composite transverse strength due to the process-induced in situ response and residual stresses. This indicates that residual stresses contribute to the scatter in the transverse mechanical properties. In order to better understand the effect of the manufacturing process, results for 100 fiber RVEs are discussed in more detail in the following section. Subsequently, a summary of the size effect study is reported in Section 3.2 to determine an appropriate RVE size for cost-efficient numerical analysis. Finally, results from a statistical analysis of all the RVEs considered in the size effect study are compared in Section 3.3 to provide direct correlation between the mechanical properties and the RVE fiber packing.

### 3.1. 100-Fiber RVE

**Figure 10:**
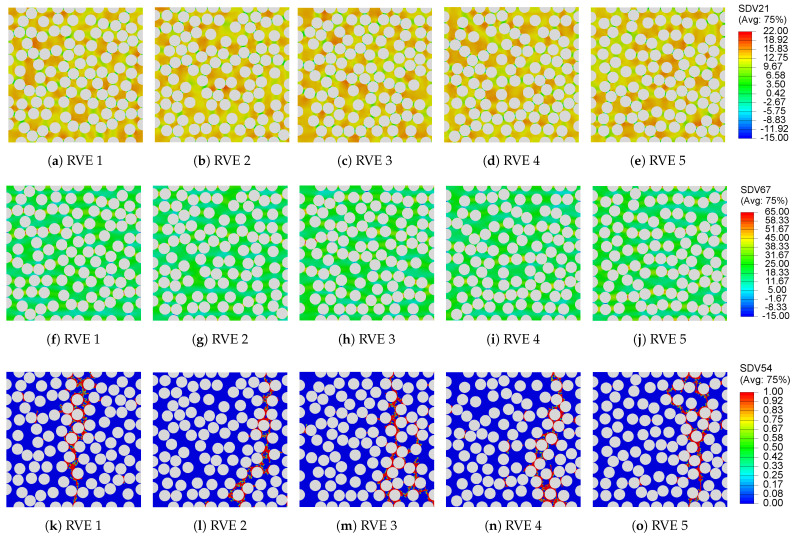
Contour plots of (**a**–**e**) process-induced residual stresses (maximum principal) obtained at the end of curing from direct process modeling analysis, (**f**–**j**) maximum principal stresses before the onset of damage, and (**k**–**o**) damage field in the RVE at the end of the transverse mechanical loading analysis.

During virtual manufacturing analysis with direct process modeling, the RVEs are subjected to the temperature profile shown in Figure 3a. Consequently, self-equilibrating residual stresses are generated. This residual stress generation is governed by the development of the in situ matrix elastic modulus, cure shrinkage and thermal mismatch between the fiber and the matrix. The evolution of the Young’s modulus as a function of the degree of cure is depicted in Figure 3b. Prior to gelation (ϕ≤0.82), the epoxy matrix is liquid and unable to sustain any stresses. Post-gelation, sufficient crosslink networks develop and the Young’s modulus increases exponentially with the degree of cure ϕ. Therefore, no significant residual stress generation is observed in the pre-gelation phase, even though the matrix experiences thermal strains during the initial heating phase and shrinkage strains during the isothermal hold. By contrast, significant residual stress generation is observed in the post-gelation phase initially due to chemical shrinkage strains in the matrix followed by thermal shrinkage strains during the cooling phase of the cure cycle. The contour plot of the residual stresses (maximum principal), at the end of the cure, in each realization of the 100 fiber RVE are shown in Figure 10a–e. The stresses in the matrix are predominantly tensile in nature, which is consistent with the past literature [19,24,25,64]. Owing to the random fiber packing, the magnitude and distribution of residual stresses in each RVE is different.

Subsequently, the virtually manufactured RVEs are subjected to transverse mechanical loading. As evident from Figure 9a, the RVEs exhibit an initial linear stress versus strain response. The drop in the stress following the linear response is associated with damage initiation in the matrix when the local stresses exceed the matrix strength $\sigma_{\mathrm{cr}}$ and the subsequent crack propagation across the RVE. The contour plots of the maximum principal stresses, before the onset of damage in the 100 fiber RVEs, are shown in Figure 10g–k. The plots clearly show regions of high stress concentration (corresponding to warmer colors) where failure initiation is anticipated. It is evident that stress localizes in regions with dense fiber packing, which suggests that fiber proximity induces stress concentration that drives failure initiation. The damage field at the end of the mechanical loading step of virtually manufactured RVEs is presented in Figure 10k–o. Although the crack propagates differently in each RVE owing to the variations in the fiber packing, the crack consistently progresses through densely packed regions of the RVE. The peak stress in the stress versus strain plot is regarded as the transverse composite strength $\sigma_{22}^{+}$, whereas its initial slope is used to compute the transverse composite stiffness $E_{22}^{+}$. The predictions vary among the five realizations of the 100 fiber RVEs, averaging at $E_{22}^{+}=7137.4\pm 43.42\;\mathrm{MPa}$ and $\sigma_{22}^{+}=25.47\pm 0.63\;\mathrm{MPa}$.

**Figure 11:**
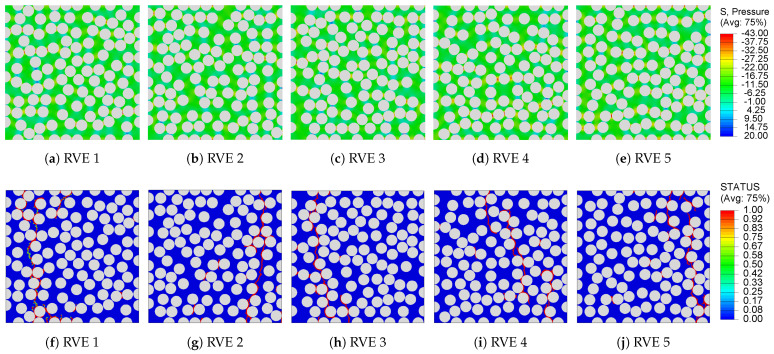
Contour plots of (**a**–**e**) hydrostatic pressure stresses before the onset of damage and (**f**–**j**) damage field in the RVE at the end of the transverse mechanical loading analysis.

For RVEs modeled with the inverse approach, the in situ matrix properties are extracted from the shear response of a $\pm 45^{{}^{\circ}}$ laminate [42]. In this set of analyses, each RVE is modeled as an initially stress-free solid. The elastic properties listed in Table 2 along with the equivalent stress versus equivalent strain plot presented in Figure 7d are used to define the behavior of a fully cured matrix subjected to transverse mechanical loads. The corresponding stress versus strain plots for 100-fiber RVEs are shown in Figure 9b. They manifest an initial linear response followed by a brief pre-peak nonlinearity. This nonlinearity is attributed to the microcracking that occurs in the laminate during the experiments [42] and is reflected in the input equivalent stress versus strain plot (Figure 7). Following the peak, the stresses in the RVE progressively drop until it has fully cracked. The contour plots of the hydrostatic pressure before the onset of damage are presented in Figure 11a–e. These plots highlight the critical regions where stress concentrates and failure is likely to initiate. A comparison with the contour plots in Figure 10f–j highlights the similarity in the local stress states before the onset of damage. However, the contour plot of the damage field at the end of the mechanical loading step, presented in Figure 11f–j, shows a different crack propagation from the direct process modeling case. This difference is attributed to the non-linearity observed in the in-situ matrix response prior to failure and the choice of failure initiation criteria defined in Abaqus/EXPLICIT [61]. The transverse composite stiffness and strength for five realizations of 100 fiber RVE are $E_{22}^{+}=7882.9\pm 44.75\;\mathrm{MPa}$ and $\sigma_{22}^{+}=25.14\pm 0.49\;\mathrm{MPa}$. These predictions agree very well with the direct process modeled RVEs confirming the robust and effective nature of the inverse analysis.

**Figure 12:**
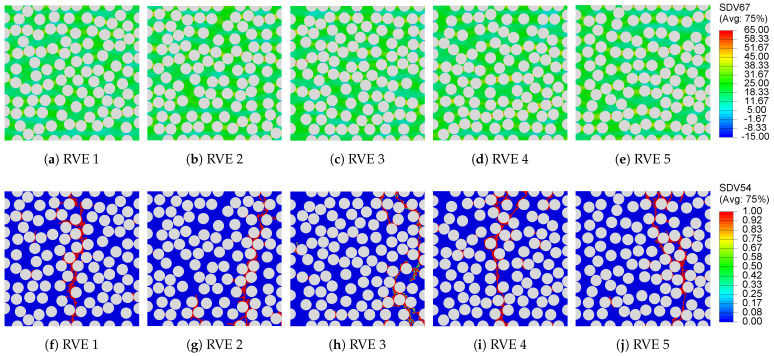
Contour plots of (**a**–**e**) maximum principal stresses before the onset of damage and (**f**–**j**) damage field in the RVE at the end of the transverse mechanical loading analysis.

The predictions from the previous two approaches are compared to a set of RVEs cured to achieve zero residual stress at the end of processing. To replicate this condition, each 100-fiber RVE is modeled as an initially stress-free, fully cured composite. The matrix is assigned material properties corresponding to the full cure state ϕ=1, as summarized in Table 2. The stress versus strain response from the mechanical loading step is shown in Figure 9c. Similar to the direct process modeling analysis, the plot manifests an initial linear behavior, which under continued loading, reaches a peak to then drop due to failure initiation until complete failure. The maximum principal stress contour plots before the onset of damage are presented in Figure 12a–e. Similar to the previous two approaches, stress concentration is observed in the fiber-dense regions of the RVE. The damage field at the end of the mechanical loading step, presented in Figure 12f–j, follows a similar path as the direct process modeling approach, suggesting that fiber distribution and stress concentration have a stronger influence over the failure in composite microstructures than the residual stress build up. The “ideal” cured 100 fiber RVEs register a transverse composite strength $\sigma_{22}^{+}=26.72\pm 1.25\;\mathrm{MPa}$, which is approximately 6% higher than the direct process modeling and inverse approach. As expected, no significant change is observed in the transverse composite stiffness prediction between process modeled (direct and inverse) and “ideal” cured RVEs. Despite that, it is clear that the presence of residual stresses contributes to the scatter in tensile transverse composite strength.

A combination of numerical approaches and precise material characterization allows to accurately quantify the influence of processing conditions on composite performance. Direct process modeling requires thermo-mechanical properties of the matrix material as a function of the degree of cure and temperature. Once these properties are obtained, process modeling methodologies allow predictions of bulk composite properties as a function of several processing conditions, enabling timely and cost-effective optimization of the manufacturing process. By contrast, the inverse approach relies on laminate testing to obtain the in situ matrix properties required for predicting the bulk composite response. Optimization with inverse approach can prove challenging and costly due to the large number of tests needed to account for each processing parameter. Furthermore, direct process modeling capabilities can be enhanced to include damage during cure, nonlinearity and viscous effects to generate a complete set of composite response under various loading scenarios. This information can then be used to design more reliable composite structures.

### 3.2. Size Effect Study

**Figure 13:**
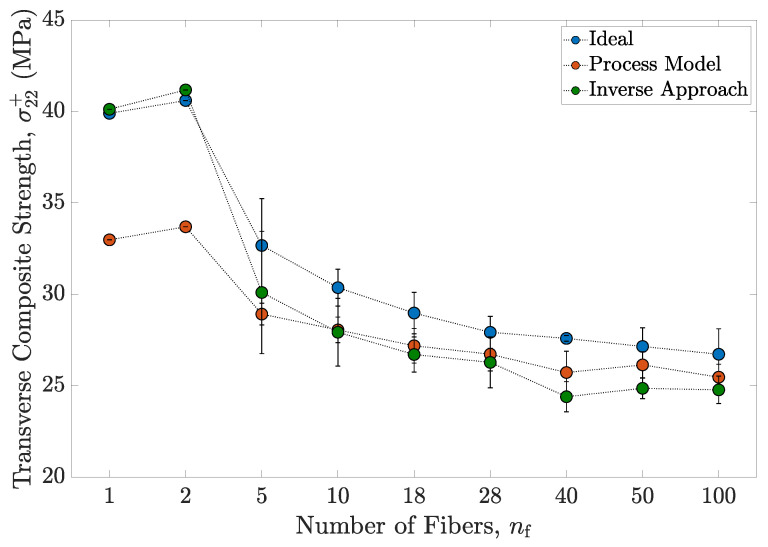
Summary of results from the size effect study showing the transverse composite strength $\sigma_{22}^{+}$ as a function of the RVE size $n_{f}$ for the three material definitions considered.

Various studies have analyzed the need to establish the appropriate dimensions of the RVEs used in micromechanical analysis [9,10,24,53,65,66,67,68,69]. Results for the size effect study conducted in this paper are discussed below. A plot showing the variation in the transverse composite strength predictions with the RVE size for the three approaches investigated in this study is presented in Figure 13. Each data point in this plot is obtained by averaging the numerical predictions from five distinct realizations for a given RVE size. The relevant standard deviations from the average value are represented by the corresponding error bars.

The RVE size manifests a strong influence on the transverse composite strength, as illustrated in Figure 13. This work confirms that fiber closeness and stress concentration, arising from random/irregular fiber arrangement, significantly affect failure in composites and therefore, the strength of the RVE [10,24,45,46,47]. Square and hexagonal packing RVEs provide overestimated transverse strength predictions [20,23,70,71]. With the introduction of random fibers, a substantial drop in the strength predictions is noticed. High stress concentrations, in areas where fibers are closely packed, act as failure initiators which result in earlier occurrences of cracking in the composite RVEs as shown in Figure 13. Figure 13 also shows convergence of strength as a function of the RVE size; the mean transverse strength prediction approaches a constant value and the associated standard deviations decrease steadily. These trends are consistent among all three approaches investigated in this study. The computational cost associated with the analysis increases exponentially with the RVE size. By considering the convergence of strength predictions as the RVE size increases, a reasonable trade-off between the prediction accuracy and the computational cost is established. For this study, RVEs consisting of 40 fibers are chosen to be a representative size. These RVEs yield a reasonable estimate of the bulk transverse properties at a moderate computational expense. RVEs of similar dimensions have been reported to provide reasonable estimates of the bulk transverse properties in [25,26,67,72,73].

### 3.3. Statistical Analysis

Comprehensive statistical analysis procedures have been established using different statistical descriptors, such as nearest neighbor distances and orientations, local fiber volume fraction distribution using Voronoi tessellation, cluster analysis with Delaunay triangulation, second-order intensity function, and fiber pair distribution function to establish RVEs geometrical equivalence [7,9,48,49,51,52,53,71,74]. In this study, statistical analysis is used to provide direct correlation between the mechanical properties and the RVE fiber packing, and to explain the standard deviation in the transverse strength as a function of the random packing. The transverse composite strength, which is shown to be very sensitive to the RVE size, is greatly affected by the random fiber distribution and close-fiber interaction. This randomness in the fiber packing can be correlated to the transverse composite strength convergence using the nearest neighbor distribution descriptor, which is based on the short range fiber interactions.

**Figure 14:**
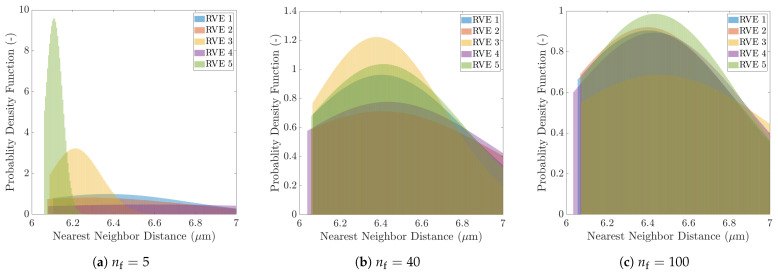
Probability density functions of the nearest neighbor distances for five realizations of RVEs when (**a**) $n_{f}=5$, (**b**) $n_{f}=40$, and (**c**) $n_{f}=100$.

The nearest neighbor distance distribution for all realizations of a 5-fiber RVE is shown in Figure 14a. For comparison purposes, similar plots are generated for the 40-fiber RVEs, which are selected as a representative model size, and the 100-fiber RVEs, the results for which are presented in previous sections, respectively, in Figure 14b,c. Each realization of the 5-fiber RVEs exhibits a distinct probability density function of the nearest neighbor distance. That is, the mean nearest-neighbor distance between several neighboring fiber pairs and their standard deviation in each RVE are highly variable. Such large variations are attributed to the small RVE size, meaning the RVE does not contain enough fibers to present converged results. By contrast, the density functions of 40-fiber RVEs, which are centered over a narrow range of neighbor distances and have similar distribution range, show better agreement. This range of neighbor distances is further reduced for 100-fiber RVEs where the density functions manifest an excellent agreement in terms of the mean distances and their standard deviations. In lieu of reducing the computational cost while achieving reasonable mechanical equivalence, a 40-fiber RVE is chosen as a representative model size for numerical analysis under transverse mechanical loading. An in-depth statistical analysis must be carried out on a larger sample set of RVEs with varying sizes for other mechanical loading conditions. Strategies for such analysis can be found in [7,9,48,49,51,52,53,71,74].

## 4. Conclusions

In the present study, the effect of manufacturing process on the transverse tensile response of composite microstructures is investigated with direct and inverse process modeling techniques. Virtual testing of cured composite microstructures results in approximately 5% variation in the average predicted composite strength and 18% in standard deviation when compared to the “ideal” cure. This suggests that the presence of residual stresses significantly contributes to the scatter in the bulk composite properties and therefore, should be accounted for during design. A size effect study, carried out to establish an appropriate RVE size, shows that RVEs consisting of 40 fibers yield a reasonable estimate of the bulk transverse properties at a moderate computational expense and are therefore chosen to be a representative size. Correlation between the randomness in the fiber packing and the transverse composite strength convergence is established for the determined RVE size with statistical analysis using a short-range fiber interaction based nearest neighbor distribution descriptor. A reasonable mechanical equivalence is observed for the 40 fiber RVE.

The potential of computational micromechanics to assess the mechanical behavior of engineering composites is presented through this study. This simulation tool, when paired with appropriate constitutive relations and accurately characterized material properties, can provide a detailed picture of the influence the manufacturing process has on the bulk composite properties. Using the direct process modeling framework described in the paper, several cure cycles can be considered and eventually tailored to achieve an optimal cure cycle to reduce residual stress generation leading to superior mechanical performance of the cured part. However, to estimate the composite response with confidence and make reliable prediction, experimental validation is necessary. This aspect is the subject of an ongoing research. Furthermore, it is seen that fiber packing has a strong influence on the composite strength predictions. An in-depth study to correlate the random fiber packing to the transverse composite strength is delegated to a future study.

## Figures and Tables

**Table 1:** Constituent thermo-mechanical properties of IM7 carbon fiber.

Property		Value	Unit
Density	$\rho^{f}$	1780	[kg/m^3^]
Axial Modulus	$E_{11}^{f}$	276,000	[MPa]
Transverse Modulus	$E_{22}^{f}=E_{33}^{f}$	19,500	[MPa]
In-plane Poisson’s ratio	$\nu_{12}^{f}=\nu_{13}^{f}$	0.28	[-]
Out-of-plane Poisson’s ratio	$\nu_{23}^{f}$	0.25	[-]
In-plane Shear Modulus	$G_{12}^{f}=G_{13}^{f}$	70,000	[MPa]
Out-of-plane Shear Modulus	$G_{23}^{f}$	7800	[MPa]
Axial Coefficient of Thermal Expansion	$\alpha_{11}^{f}$	−0.54 × 10${}^{-6}$	[K^−1^]
Transverse Coefficient of Thermal Expansion	$\alpha_{22}^{f}=\alpha_{33}^{f}$	10.08 × 10${}^{-6}$	[K^−1^]
Thermal Conductivity	$k^{f}$	5.4	[W/m-K]
Specific Heat	$c_{p}^{f}$	879	[J/kg-K]

**Table 2:** Constituent thermo-mechanical properties of RIM R135/H1366 epoxy resin for direct process modeling and inverse approach.

Property		Value	Unit
Density	$\rho^{m}$	1200	[kg/m^3^]
Direct Process Modeling			
Coefficient of Thermal Expansion	$\alpha^{m}$	61 × 10${}^{-6}$	[K^−1^]
Chemical Shrinkage Coefficient	$\beta^{m}$	0.111	[-]
Thermal Conductivity	$k^{m}$	0.245	[W/m-K]
Specific Heat	$c_{p}^{m}$	1600	[J/kg-K]
Elastic Modulus	$E^{m}$	2482	[MPa]
Poisson’s ratio	$\nu^{m}$	0.37	[-]
Critical Strength	$\sigma_{\mathrm{cr}}^{m}$	64.1	[MPa]
Fracture Toughness	$G_{\mathrm{IC}}^{m}$	0.001	[J/m^2^]
Inverse Approach			
Elastic Modulus	$E^{m}$	2956	[MPa]
Poisson’s ratio	$\nu^{m}$	0.37	[-]
Hydrostatic Cutoff Stress	$p_{\max}^{m}$	43	[MPa]

**Table 3:** Experimentally determined cure kinetic constants for RIM R135/H1366 epoxy resin.

Property		Value	Unit
Exponents	*m*	0.4	[-]
	*n*	1.5	[-]
Rate Constants	$A_{1}$	3.6 × 10${}^{9}$	[s^−1^]
	$A_{2}$	0.01245	[s^−1^]
Activation Energy	$\Delta E_{1}$	85.3	[kJ/mol]
	$\Delta E_{2}$	11.1	[kJ/mol]

## Data Availability

Not applicable.
